# From stakeholders to protagonists: an exploratory framework for cultivating prosocial capacities for development

**DOI:** 10.3389/fsoc.2024.1199755

**Published:** 2024-11-06

**Authors:** Darren Kirk Hedley

**Affiliations:** Department of Anthropology and Archaeology, University of Calgary, Calgary, AB, Canada

**Keywords:** global south, participation, capacities, empowerment, governance, prosocial, altruism, stakeholders

## Abstract

The world in 2024 faces numerous interlinked crises, including climate change and water shortages, rising geopolitical tensions, and a new awareness of the risks of pandemics. These crises reverse decades of incremental development progress and humanity’s aspirations embodied in the 2030 Agenda for Sustainable Development, necessitating a more active and collaborative participation of development stakeholders. The magnitude of challenges points to the need for transformational approaches to releasing the potential of stakeholders, which requires building on and extending beyond current best practices in participation and capacity strengthening. What is most needed today is a balanced assessment of the complexity of human nature and a vision that recognizes the *prosocial* potential of people to harmonize the pursuit of personal interests with a willingness to contribute to social and collective development goals. Prosociality is a capacity that all stakeholders can strengthen—from individuals to institutions to communities (including different forms of social groupings). These stakeholders can become empowered as active *protagonists* of development, with the potential to work synergistically to achieve the Sustainable Development Goals (SDGs). In order to work with these protagonists, it is important to view them systematically in terms of key characteristics such as their antecedent knowledge, values and culture, stance, agency, roles, relationships, and learning.

## 1 Introduction: the need for prosocial protagonists

The international community has achieved a considerable alignment of efforts for poverty reduction and development, through such means as the Milennium Development Goals and the current Sustainable Development Goals (SDGs) within the 2030 Agenda for Sustainable Development.^1^ Despite significant progress toward these goals, the world in 2024 has seen major setbacks caused by 3 years of the COVID-19 pandemic, the Ukraine war, and climate related crises. Now halfway to 2030, the SDGs “are disappearing in the rear-view mirror, as is the hope and rights of current and future generations.” (United Nations, 2024a). Thus, while SDG 6 calls for achieving safe and affordable drinking water, sanitation, and hygiene for all by 2030, billions of people will still lack access to these services unless the rate of progress increases by 300% for hygiene, 400% for sanitation, and 600% for drinking water (United Nations, 2024b). SDG 15 calls for halting biodiversity loss, but this is threatened by the increasing toll of deforestation, desertification, land degradation, and loss of natural habitats (United Nations, 2024c). There is also little optimism about the world’s ability to reduce greenhouse gas emissions and limit temperature change to the 1.5°C called for by SDG 13 and the associated Paris Agreement (United Nations, 2024d). As stated by UN Secretary-General António Guterres: “We must rise higher to rescue the Sustainable Development Goals—and stay true to our promise of a world of peace, dignity and prosperity on a healthy planet” (Guterres, 2022).

This paper suggests that a fresh approach is needed to make further global development progress, and two ideas could be potentially transformative in this connection. The first idea is *prosociality*—as part of the complexity of human nature, people do possess a *prosocial* orientation toward social or wider goals, alongside or in preference to personal or more circumscribed aims. The second idea is that a prosocial emphasis should be simultaneously promoted within individuals, communities, and institutions, three interrelated categories of stakeholders who can become empowered as the *protagonists* of development. This introduction provides an explanation of why an approach to prosocial protagonists is needed, and the paper then provides background on participation and capacity strengthening, followed by an in-depth discussion of the ideas of prosociality and protagonists. It concludes with a proposed framework for analyzing the dimensions of protagonists in such a way as to facilitate the promotion of prosociality among them.

Some will look at the current stagnation in development progress in terms of the need for more funding support, and while more resources are necessary, an emphasis should be placed on empowering and aligning various actors in economic, political, and social spheres. Each protagonist brings their own resources to the development process—financial, expertise, networks, human resources, and motivation for change—and much potential can be unleashed by harnessing those resources. Undoubtedly, an unequal global economic and political system oppresses and restricts the engagement of many. Reaching development goals requires empowering the collaborative participation of a range of actors, from the households and community institutions that take initiatives and advocate for improved services, to the donors, policy-makers and managers that fund and implement development programs. Our primary interest is with actors in the Global South, but their development path is intertwined with the actions, perceptions, and support of Northern countries—whose development is also included in the SDGs.

Participation is already mainstreamed in poverty elimination programs, and it is recognized as being essential to the achievement of the United Nations 2030 Agenda. For example, SDG 16 seeks to “promote peaceful and inclusive societies for sustainable development, provide access to justice for all and build effective, accountable and inclusive institutions at all levels.” Other targets specify the participation of women (target 5.5) and developing countries (target 10.6), and participation is a sub-goal of sectoral targets such as 6.b, to “support and strengthen the participation of local communities in improving water and sanitation management” (United Nations, 2024e). Still, the conditions for facilitating, empowering and encouraging participation may be elusive. The UN can itself at times display tendencies contrary to genuine participation, such as an inordinate influence by corporations or by Western powers, which at times bypass or starve the UN of finances necessary to carry out its mandate (Sogge, 2021). There is a risk that participatory development approaches sometimes become ritualistic and even “tyrannical” (Cooke and Kothari, 2002), which has led to calls to make those efforts “transformative” (Hickey and Mohan, 2004). It is encouraging that participatory approaches continue to evolve, linked to empowerment, strengthening governance and the defense of human rights, and these advances are discussed in section two of this paper.

Despite the evolution of ideas about participation, there is insufficient discussion about the complexity of human nature, the motives of those that participate, and the possibility to leverage people’s full capacities to contribute to the advancement of society. The term prosocial is taken from Atkins et al. (2019) to refer to a behavior pattern emphasizing getting along with others and acting so as to benefit the community or society as a whole. Prosocial behavior includes altruism, a concept which implies some degree of self-sacrifice, but the prosocial definition is broader to include all types of behavior in which mutual benefits are sought, including “win-win solutions.” Without entering into definitional details at this exploratory stage, to be prosocial broadly means being cooperative, service-oriented, caring and considerate of others, peaceful, ethical, or moral.

Placing greater emphasis on strengthening prosocial capabilities is crucial for promoting participation, but it raises the question of who is included in that participation. Individuals participate in their own personal and societal development, but their beliefs and attitudes are shaped by communities and social groups, while institutions establish the frameworks and policies that either enable or limit their participation. Furthermore, institutions can either lead transformative change or hinder development, making it essential to strengthen their capabilities and policies for improved governance and the sustainability of beneficial innovations. This paper presents elements of a conceptual framework for the prosocial participation of three categories of protagonists of the development process—individuals, communities, and institutions. While terms like stakeholder can have a more passive connotation, the word *protagonist* implies a more active connotation, with an orientation toward change. Development programs often target certain groups of individuals or organizations, but in reality the protagonists are highly intermeshed and mutually influential, and efforts to strengthen prosociality should generally consider them more holistically.

As one example, corruption is a problem that shows a deficit of prosociality, which impedes development progress through the loss of trillions of dollars of public procurement resources (World Bank, 2020) and the erosion of cohesion and trust. For years it was assumed that individual officials with power to control a service or good would use that power for personal rather than public interests, unless they are held accountable (Klitgaard, 1998). This led to a mainstream approach of anti-corruption strategies, action plans, laws, and training, which have not yielded appreciable results (Disch et al., 2009). Today, there is a more widespread recognition that corruption needs to be reduced by strengthening integrity as an individual behavior and a systems characteristic. Spector (2022) draws on decades of anti-corruption experience to conclude that more needs to be done to address the embedded ethical, social, cultural, and psychological frameworks that determine the balance between corruption and integrity, including modes of socialization and extrinsic and intrinsic motivations. Many practitioners of anti-corruption programs now seek to mobilize various stakeholders, including civil society, the private sector, and the mass media for greater transparency and accountability in service delivery (Water Integrity Network, 2021).

A more subtle application of these principles regards the limitations on the willingness and ability of people to voluntarily participate in development programs for the benefit of their own communities, an issue which the author has witnessed in the course of implementing and evaluating many development projects. Volunteering occurs spontaneously within all cultures and in many planned interventions, but the experience of development programs is that people are often hesitant to contribute their time to take initiative, help manage services or hold institutions accountable. In some cases, this may be attributed to projects having been imposed by external government or nongovernment agencies with limited local ownership, but the pattern is often found even when considerable efforts are made to establish a partnership approach from the earliest design stages. Critical observers may point out the irony of paid agency staff apparently setting the terms for interactions with poor, unpaid community members, and it is clear that participation by the poor (especially women) can be constrained due to a lack of time or other factors (Banerjea, 2011). In response to these types of concerns, some development agencies have sought to ensure greater local control, downward accountability, and adjusted expectations of volunteers, while seeking to financially support longer-term and time-intensive roles of local individuals through means such as community or state-generated allowances. While there are often incentives for managers to pragmatically focus on the short-term needs of a development project when resolving such challenges, permanent arrangements are needed to optimize roles and relationships among protagonists.

Given the various obstacles to achieving the SDGs, a fresh and visionary restart is needed—what the UN Secretary-General has called “a new social contract for a new era,” an evolving agreement between the people and the state, incorporating new norms, systems, and structures (UN Volunteers, 2022). This paper advocates a more fundamental look at our assumptions about people’s motivations and the basic pattern of relationships among protagonists. We may make observations about current realities and draw conclusions based on that, but individuals, communities and institutions are amenable to influences, and they can and do change. As we move forward in the coming years and decades, we will need to monitor the evolution of human capacities, and seek out new mechanisms and roles for citizens, communities, and institutions to function within those societies.

### 1.1 Scope and structure of the paper

At this juncture, several clarifications are required about the terms used, the geographical and disciplinary focus of this paper, and the intended readers. While the paper centers around the empowerment of all development protagonists, it is expected that readers will be practitioners and academics who—it is hoped—can ultimately influence development policies in favor of prosocial empowerment. The paper is inspired by the challenges of the water and environmental sectors, though the central argument is applicable to all sectors and the approach is interdisciplinary. The gravity of the crisis facing humanity calls for a willingness for professionals to step outside their comfort zones to explore new ideas with a wider range of stakeholders, and the paper bridges the gap between global and local thinkers and actors.

Another gap exists between “positive practitioner” and “negative researcher” cultures (Chambers, 1983), and the author is mindful of this based on his background as a program manager, evaluator, and academic. The conceptual framework advanced here draws considerably on the author’s experience of the challenges in current development approaches and promising lines of research and action. As such, the paper advocates a more conscious *analysis* of societal conditions and the way that frames shapes our discourses and our solutions (Fischer, 2003), but it also leans toward *prescription* by broadly outlining policies and approaches for more effectively promoting participation for sustainable development.

The endpoint of this paper is a preliminary conceptual framework centered around the concept of prosocial protagonists. A conceptual framework was explained by Rapoport (1985, 256) as follows: “…models describe how things work, whereas theories explain phenomena. Conceptual frameworks do neither; rather they help to think about phenomena, to order material, revealing patterns—and pattern recognition typically leads (thereafter) to models and theories.” The paper will argue that our framing and discourse around stakeholder engagement can limit and channel our thinking in certain ways that are not conducive to optimal engagement of actors in development. As such, this framework is intended to help analyze the interactions of actors, and to inform action and research. Whether we are working within such a development setting, or looking from a distance and thinking about the roles and relationships, of actores, it is hoped that this conceptual framework will help generate some fresh thinking for better development results.

Given the focus on developing a conceptual framework to help strengthen prosociality among protagonists for global development, the paper does not delve into the contributions of various disciplines, or examine details of specific training and educational approaches for promoting prosociality. Much of prosociality obviously emerges during childhood, from family and school education to the significant influence of the media (Piotrowski et al., 2015). As stated by a Honduran water sector official:

*These values such as honesty and integrity are part of the education of every individual from their childhood. It is difficult to behave with integrity if you do not have seemly principles and values, if you do not have moral values as part of your personal education, you wont easily change your behavior as an adult, or adopt ethical behavior if you become a public official* (Espinal, 2020).

It will remain for future papers to further develop such lines of inquiry.

The paper is structured as follows: Section 2 reviews the cutting edge of mainstream human development concepts surrounding participation and capacity strengthening, ideas which are essential to build on while searching for a more transformative approach. Section 3 advocates a view that recognizes the prosocial capacity of protagonists to contribute to collective as well as individual goals, and it outlines an approach to working with that potential. Section 4 builds on these ideas to explore elements of a conceptual framework of the three categories of protagonists, in terms of key characteristics such as antecedent knowledge, values and culture, agency, stance toward other stakeholders, roles, relationships, and learning.

## 2 Current concepts of participation and capacity strengthening

As previously mentioned, the manner in which development agencies assess problems and determine approaches should take a flexible and evolving measure of the capacities of protagonists to help them participate in the development process, and this requires some review of current thinking in the field. First, this section discusses the concepts of participation, empowerment, and governance, to help prepare a more nuanced vision of protagonists participating in various program and policy spaces. Then, concepts of capacity strengthening, capabilities, human rights, and social capital are introduced, to complete the picture. It is important to build on progressive thinking in these areas as an important foundation for assessing people’s prosocial potential and working effectively with development protagonists, as suggested in the framework of protagonists to be elaborated in subsequent sections.

### 2.1 Participation, governance, and empowerment

Participation is introduced here in conjunction with empowerment and governance, because participation both presupposes and reinforces the empowerment of protagonists in the development process—which includes the governance of local, national, and international institutions. *Participation* may be defined simply as people being involved in decisions that affect them (Thorpe and Apgar, 2022), especially those at the grassroots level that have less voice and power in the international political economy. Chambers points out, however, that the common phrase of “handing over the stick” to local people implies not just giving them a voice in development, but more generally handing over responsibility, initiative, control and power. Participatory methods include facilitating and enabling marginalized people to engage in development program appraisal, research, planning, action, monitoring, evaluation, facilitation and other activities (Chambers, 2017).

This requires greater clarity in visualizing the development processes in which we expect participation to be applied, and as such there are two fundamental views: *immanent development*, which is simply the natural progress of societal evolution, and *intentional development*, which entails the specific activities of institutions aimed at improving social, economic, and political conditions (Thomas, 2021). In this regard, participation is promoted in specific “development” interventions, but also in normal social, economic, and political life. The justification for greater public participation is often stated in normative terms, such as increased empowerment and emancipation, while at other times functional and instrumental considerations are given more weight (Coenen, 2009). The latter considerations place emphasis on increased legitimacy of decisions, reduced conflict, better information to analyze problems and assess options, and enhanced learning by people which encourages them to change their behavior. Another distinction has been made between participation as a means to an end, such as mobilization of a population toward a short-term project, and participation as an end in itself—as a long-term process of strengthening the capacities of people to directly intervene in development activities (Oakley, 1991). This paper considers participation in both immanent and intentional senses, from the standpoint of both the normative and functional justifications, and as a means to development but more importantly as an end in itself. In order for this deeper participation to take root, it not only requires active efforts by protagonists and development agencies, but also the removal of obstacles and structural impediments that have made it more difficult for countries and individuals in the South to naturally follow their own development path. These factors include hurdles to accessing technology and financing with reasonable terms, and the control over common resources by a small number of players who have inordinate influence over the rules of the economy (Dixson-Decleve et al., 2022).

Of course, as stated previously, not all of what is called “participation” actually allows for the free expression of views and the sense of collective responsibility and ownership that it usually implies. Also, participation is not a formula, and the type, timing, and amount of participation may need to be carefully calibrated to each situation (Hurlbert and Gupta, 2015). For example, there are differences between the degree and type of participation of mothers in a child health care group, and citizens in a large infrastructure project. Given the centrality of participation in the development agenda, the means of promoting it has been given much attention, and there are numerous written guidelines to this effect (e.g., Bopp and Bopp, 2011). NGO projects, for example, are expected to have high standards of participation; they have, consequently, established guidelines to apply in a growing set of circumstances, such as for water, sanitation, and hygiene (Oxfam, 2020) and in emergency responses (Groupe URD, 2009). The project parameters that can facilitate participation—such as having adequate time and funding—are also crucial to consider, and, on this point, it must be acknowledged that donors play a major role in enabling or limiting participatory practices. Promoting participation in national government services may be more limited, but, for example, a World Health Organization handbook describes participation as a key element of a social contract for universal health coverage, one entailing dialogue between service providers and users (WHO, 2021).

One of the newer approaches to participation is through the use of online tools and digital communication technologies, such as websites, social media, and teleconferencing and messaging applications. While these show considerable potential, genuinely empowering people to participate also requires ensuring they have knowledge and skills to use these spaces effectively. Rather than being delivered through a simple technological fix, participation requires broader capacity strengthening through a range of measures that build collaboration and shared responsibility. For example, as found in a global evaluation of COVID-19 response projects, participation in such projects is more effective when Northern-based implementers have long-standing partnerships with local organizations and communities (TANGO International, 2020; TANGO International, 2022). Among other observations on the current state of participatory practices, it is clear that as new circumstances and capacities emerge in the coming years, there needs to be ongoing research on how to promote participation and collaboration among protagonists.

*Governance* is a crucial concept, which embodies the aspiration for broader and more profound participation in government and having a voice in shaping development projects, programs, and policies. The concept also acknowledges that decision-making evolves through the ever-changing roles of, and relationships among, stakeholders. According to the Commission on Global Governance:

“*Governance is the sum of the many ways individuals and institutions, public and private, manage their common affairs. It is a continuing process through which conflicting or diverse interests may be accommodated and co-operative action may be taken. It includes formal institutions and regimes empowered to enforce compliance, as well as informal arrangements that people and institutions either have agreed to or perceive to be in their interest*” (Commission on Global Governance, 1995, p. 3).

Importantly, this definition also highlights that “informal arrangements” can be considered as part of governance, and in this paper, informal actors are categorized as part of the community which has a role in governance. At the same time, community-level formal institutions also play a role. Citizen roles can include more than simply voting and paying taxes. They can help, for example, in carrying out situation analyses and needs assessments, providing input to policies and investment plans, and giving feedback on the implementation of plans.

*Empowerment* is a term which helps with conceptualizing the necessary conditions for equitable and genuine participation of people in all aspects of society, from making decisions, to taking action, to contributing to knowledge. Perspectives on empowerment are varied, and for example the term is often used to imply a process of change in individuals who lack power, resources and personal capacities. Others are critical of the focus placed on changing poor people, arguing that there is a need to change the structures and institutions of economic and political power in which the poor are embedded (Veltmeyer, 2021). Empowerment can be seen as a fuzzy term, lacking clear boundaries and tolerant of uncertainty (Uddin, 2012). Kabeer states that many feminists find the “fuzziness” of empowerment to be advantageous during a process of learning, citing an Indian activist:

“*I like the term empowerment because no one has defined it clearly yet; so it gives us a breathing space to work it out in action terms before we have to pin ourselves down to what it means. I will continue using it until I am sure it does not describe what we are doing*” (Kabeer, 2001).

Still, the following definition is helpful: “Empowerment thus refers to the expansion in people’s ability to make strategic life choices in a context where this ability was previously denied to them” (Kabeer, 2001). Beyond this working definition, it is useful to categorize models for empowerment of women and other vulnerable groups, in terms of: *power over*, such as when there is a conflict involved; *power within*, by activating personal assets like self-esteem; *power to*, such as in learning to read; and *power with*, referring to collective action (Mosedale, 2005). With this in mind, empowering participation requires removing obstacles to the inclusion of marginalized groups and releasing the potential power and agency of individuals, communities, and institutions.

### 2.2 Capacity strengthening, capabilities, human rights, and social capital

Participation as discussed above requires an augmentation of the power of protagonists in various senses of the word, and gradually strengthening people’s capacities, which international agencies have a responsibility to foster. Human rights and social capital frameworks are referred to here as useful ways to channel these efforts; the concept of social capital is a good example of recognizing the inherent capacities of protagonists to invest and take the lead in their own development process. Capacity building is a frequently used term, but this may be taken to imply a process of starting from scratch, thus it is now seen as more accurate and respectful to speak of *strengthening* or developing capacities already in place (LenCD, 2022). A popular definition of capacity strengthening is provided by the UNDP: “the process through which individuals, organizations and societies obtain, strengthen and maintain the capabilities to set and achieve their own development objectives over time” (Zamfir, 2017). The 2005 Paris Declaration on Aid Effectiveness emphasized that donors should play a supporting role to “partner” countries to strengthen capacities to analyze, plan, manage, implement, and account for results of policies and programs (Zamfir, 2017).

Approaches to capacity strengthening vary, and ongoing critique and reflection has generated many lessons, among which is the observation that development agencies often need capacity strengthening themselves (Eade, 2007). Chambers (2017) finds that participatory approaches are more likely to encourage greater humility and recognition of errors and biases by implementers, thereby leading to better mutual learning. Northern organizations can unwittingly insist on their cultural style of procedures, which may privilege Northern actors and limit capacity building. Capacity development efforts must consider the complex political realities of each situation, such as the advantages selectively enjoyed in the status quo and how these would be affected by shifts in roles and responsibilities. Issues of class structure and political stability also arise, and communities need to be empowered to allow for an engaged society that can hold government and other agencies accountable (Venner, 2015). Some mainstream approaches now consider that capacity strengthening of an organization should necessarily be pursued within a complex ecosystem (USAID, 2018), and the 2009 UNDP capacity development primer advocates work on the level of the individual, the organization, and the enabling environment (UNDP, 2009). By using and transparently sharing theories of change, protagonists can be enabled to help guide programs (Ortiz Aragón, 2010). This idea of capacity strengthening in an interconnected way is an important aspect of the prosocial protagonists framework.

The idea of *capabilities* crystallizes capacity in a framework that accommodates individuals, communities, and institutions. Capabilities have been elaborated by Sen and Nussbaum as providing the freedom for people to lead the life they want. Sen writes about functionings as the various things a person may value doing, from meeting their elementary needs of nourishment and health, to more complex activities such as taking part in the life of the community. Capabilities comprise the set of alternative functionings that an individual can choose from (Sen, 1999). This resonates with the idea of protagonism advanced in this paper, as a condition in which individuals can voluntarily exercise their volition regarding the achievement of their needs and wants. Nussbaum adds that internal capabilities are not just inside a person but they also reflect the opportunities created by a combination of personal abilities and the political, social, and economic environment (Nussbaum, 2011). Nur University, discussed below, will be used to demonstrate a framework of capabilities pertaining to personal, interpersonal, and social transformation which has been fostered on a large scale (Anello et al., 2020). While the word capability has an additional meaning in some quarters, this paper will generally use the more common term “capacities.”

The capabilities approaches of Nussbaum and Sen are seen as a species of *human rights* approach, and a human rights lens also provides greater emphasis on the fundamental social contract in which society fulfills the basic conditions of life for all citizens, with all social and civic rights understood as being inseparable and inalienable. The other side of the contract is stated in a 1998 UN General Assembly declaration which confirmed that “everyone has duties toward and within the community, in which alone the free and full development of his or her personality is possible.” Specifically, “individuals, groups, institutions and non-governmental organizations have an important role to play and a responsibility in safeguarding democracy, promoting human rights and fundamental freedoms and contributing to the promotion and advancement of democratic societies, institutions and processes” (UN General Assembly, 1998). With the acceptance by duty-bearer nation-states of this obligation, there is an imperative to progressively realize all of these rights, using the maximum of their available resources and fulfilling the minimum core obligations (OHCHR Special Rapporteur, 2020). The principle of progressive realization of rights coincides with the vision of evolution toward an ideal of prosocial protagonists.

*Social capital* is another way of expressing capacity strengthening in communities, taken to include relations of trust and reciprocity, the establishment of norms, and strengthening a sense of connectedness (Steiner and Hanks, 2016). One implication is that social capital creates a type of resource that can make development efforts extend further with greater quality and sustainability. It is important to take a wider view of its implications, to avoid instrumentalizing social characteristics as a means to achieve economic goals (Claridge, 2018), In this regard, social capital can be seen in three levels: bonding social capital, which captures connections between community members; bridging social capital, which involves connecting one community or group with other communities/groups; and linking social capital, which refers to networks across formal boundaries, such as links to government authorities (Smith and Frankenberger, 2018).

It is clear that the current discussions of participation, capacity strengthening, and related concepts have led to important insights that can inform humanity at this critical juncture and inform the nature of collaboration that protagonists can have. A key concept that has been less obvious or explicit in most of the literature on these subjects is that of the prosocial orientation of protagonists, and these are essential conditions to help unlock the potential resources that protagonists can deploy and invest in development.

## 3 Prosociality

This section discusses the complexity of human behavior and asserts the potential to strengthen people’s prosocial capacity. The argument advanced here is that global crises and stalled progress on development goals necessitates a more concerted and transformative approach to capacity strengthening and participation; it would seem naïve to think that by continuing the same strategies that have been used for decades, humanity will be able to meet the needs of today. A relatively new strategy would be to broadly focus on promoting greater prosociality, which could be applied in such ways as voluntarism, cooperation, mutual aid during emergencies, and strengthened integrity and resistance to corruption. Prosociality should be seen as a balancing of people’s natural responsibility for themselves and their families, while they show concern for and help serve their communities, and feel a shared responsibility for society. It should be noted that prosociality can be a characteristic of communities and institutions, in addition to individuals, but the discussion in this section focuses on individual protagonists.

Admittedly, self-interested competition and excessive consumption are all-too-visible in human affairs, leading to a one-sided perception of human nature. As a result, policies and practices tend to be shaped around an assumption of the stereotype of *homo economicus*, who will always prioritize personal benefits with little regard to societal impacts and needs. People tend to have implicit assumptions about this, based on what stands out in their memories of their own experiences. But as we will see below—prosociality is both a characteristic of individuals and a perspective through which the observer perceives other individuals, and efforts to strengthen prosociality would need to address both.

A narrow focus on current anti-social and self-serving behavior is not conducive to building capacities for a broader and more consistent participation of protagonists. Development programs commonly diagnose a lack of some resource or capacity, and aim to strengthen that, and prosociality should be seen in the same light. While it is necessary to analyze and work with the complexity of current conditions, it is suggested in this paper that we should be open to the possible evolution and strengthening of prosociality. In order to perceive and work with the prosocial potential in human behavior, one requires the faculties of:

1. *recognizing* that prosocial behavior is a part of human nature, and
2. *embracing the complexity* of human nature, by working constructively with the mixture of self-serving and collaborative behaviors that are found.

### 3.1 Recognition of prosocial behavior

A positive orientation toward prosocial potential depends on the faculty of sensitivity to and awareness of prosocial behaviors and patterns in society. The assertion made here is that such recognition is made possible when a person has:

- awareness of prosocialism as an ideal;
- reflectivity about what guides the behavior and assumptions of oneself and others;
- an understanding of how language and discourse shapes our thinking;
- openness to examples of prosocial *individual* human behavior; and
- openness to the evidence of *collective* human progress.

#### 3.1.1 Ideals

First, protagonists need to have an awareness of the *centrality of ideals* in human development, and a *sense of hope* that humanity can draw on these ideals to overcome our current crises. It is reasonable to presume that at least partly, societies have historically been able to survive and advance because of people’s abilities to care for and coordinate with others for the sake of the society, and that this tendency has balanced out or exceeded our self-centered or destructive tendencies. This idea is found in the historical survey of Kropotkin (1902), who highlights mutual aid as a fundamental factor of human evolution. Rochester holds that a positive revolution is within our reach, and he is optimistic about the future of humanity, stating:

“*…it is not wishful thinking that leads us on, but an ever so faint ray of hope that that which is not entirely impossible will emerge as real*” (Rochester, 2018).

The ideal of interconnectedness is common in cultures around the world. In South Africa, a country renowned for rising above past conflict, the *ubuntu* ethic from the Nguni people holds that one’s aim in life should be to become a genuine person, to realize our higher, human nature (Metz, 2021). As stated in a children’s book on Ubuntu:

“*When I look into your eyes, I see myself. I am you … To be kind to you is to be kind to myself. I am you, and you are me. I am because we are one*” (Moahloli, 2022).

Laszlo argues that humanity needs a star to follow and a vision of positive ideals, and that both the ideals of world religions and modern ethical worldviews embody the perennial values needed in this regard. These include the Christian vision of universal brotherhood; Islam’s vision of a community of God, man, nature, and society; and Buddhism’s focus on humanity’s joining in the interrelatedness of all reality by rejecting the desires of the ego. The more recent Bahá’í Faith promotes a world-embracing vision of an ever-advancing civilization based on peace and justice. Laszlo goes on to assert, however, that over time, the positive visions of religions become “encrusted with obsolete practice and ancillary beliefs,” such that religion has lost its influence. Yet, he also states that there are limitations in the new visions that have replaced them, such as those of Marxists and liberals (Laszlo, 1989), which have a narrow emphasis either on structural change or rational self-interest, respectively.

Similarly, Durkheim had noted that religious conceptions long served as the vehicle for expressing essential moral ideas, and he advocated the identification and promotion of the rational essence of these ideas in modern society even when delinked from religion. He saw our allegiance to society as a single psychic entity or “moral being” to which our individual wills can be linked (Durkheim, 2002), a conception which Bellah considered close to divinity (Tiryakian, 1974). Durkheim promoted moral education, and he wrote that true morality arises from autonomous individuals acting freely to sacrifice for the benefit of those beyond one’s circle of self-interest. Loyalty to family, nation, and humanity are consecutive stages of our social and moral evolution, and:

“*If one loves his country, or humanity in general, he cannot see the suffering of his compatriots—or more generally, of any human being—without suffering himself and without demonstrating, consequently, the impulse to relieve it*” (Durkheim, 2002).

While people will have differing opinions when it comes to the source of ethics and morality, many are coming to realize that our survival depends on strengthening hopeful visions of human potential and high ethical standards. This should logically be reflected in what is taught in schools, and for example the OECD Learning Compass 2030 sets out a learning framework to prepare students with transformative competencies to cope with trending conditions including climate change, with a clear focus on both individual and collective well-being (OECD, 2024). The revolution needed today is one involving substantial changes in relations among people, and between people and the environment. For example, Lakoff’s vision of a New Enlightenment includes an ecological consciousness that is spiritual, which derives from our deep connections to the natural world and to each other. This consciousness, he asserts, helps inspire a sense of morality, gratitude, and awe as comprising the fundamental basis of reason and action (Lakoff, 2008).

Many from a faith-based background acknowledge the value of all moral and ethical frameworks, but they maintain nonetheless that a purely materialistic approach to development is insufficient to elicit heartfelt compassion and draw on the depths of human ethical capacities. Writing as part of a group of faith-based scientists convened by the International Development Research Center, Arbab (2000) points out that the vast majority of humanity considers spirituality to be their main reference for meaning and motivation. If they are to be protagonists in a process of development, their cultures, beliefs, and values should be granted their due respect and place. In addition, he argues that science and religion can be seen as complementary systems of knowledge, while spirituality is essential for building unity and commitment to achieve development and establish authentic justice (Arbab, 2000). Indigenous spirituality is of particular relevance in this connection (OHCHR, 2022), partly because there is increasing recognition of indigenous spirituality and worldviews as providing insights and inspiration for grappling with environmental crises (United Nations, 2024b).

#### 3.1.2 Reflectivity

The second aspect of the faculty of recognizing prosociality is an understanding of the importance of *reflective thinking* on our personal values, strengths, and weaknesses. Reflective thinking can help achieve balance in our knowledge of ourselves, our communities, and institutions, and to accurately assess the conditions and prospects for future action. In the organizational learning field, Argyris and Schön (1996) describe single-loop learning as a cycle of asking instrumental questions that assess performance within established strategies, while double-loop learning proceeds through a wider cycle of questioning around the underlying theory and assumptions behind those strategies. Such reflection can also help us gain perspective on how our thoughts and actions are structured by what are variously described as paradigms, mental models, discourses, narratives, or frames.

The practice of moral education often involves reflectivity in clarifying one’s own values and learning to make decisions based on our values and their application, with open-minded reason. Kohlberg’s approach to morality neither posits a set of universal moral norms nor considers morality to be exclusively an individual choice. Instead, he promotes a higher form of moral thinking, one that transcends personal fear of punishment or societal expectations, and focuses instead on conscience, principles, and ideals of justice (Chazan, 2022).

#### 3.1.3 Language, discourses, and framing

The third aspect of the faculty of recognizing prosociality is the perception of the influence of *language and discourse* in augmenting our perception of the pervasiveness of self-serving, conflictual, and competitive behavior. These shape the observer’s view of the social-political world, rather than merely mirroring it, and this has implications for the way we act in the world (Fischer, 2003). Karlberg writes that the phrase “that’s just human nature” may seem like a simple linguistic expression, but it brings a culturally coded set of meanings and practices along with attitudes and beliefs that the pursuit of self-interest is inevitable. For example, Hobbes encouraged aggressivity in European and American thought with his metaphor of a “war of all against all” to characterize human societies, while Confucius’s description of societies as extended families was a more harmonious influence to Chinese thought (Karlberg, 2004).

Metaphors like these often arise from oversimplifications of complex ideas in various fields, and become part of a narrative that can shape our thinking. In evolutionary theory, the narrative which emerged from the metaphor of the survival of species became fixated on the individual’s struggle for survival, although Darwin’s original use of the metaphor in fact also highlights symbiosis and the interdependence of individuals within societies (Lakoff, 2008). In economics, a one-sided reading of Adam Smith focused on the image of rational economic man (Raworth, 2017), while overlooking that Smith also wrote:

“*How selfish so ever man may be supposed, there are evidently some principles in his nature, which interest him in the fortune of others, and render their happiness necessary to him, though he derives nothing from it except the pleasure of seeing it*” (Smith, 1759).

Not only do these ideas about human nature influence our perception of others, but this can translate to our own behavior. Raworth (2017) cites research by Frank with economics students, which demonstrated that they become more self-centered as they progressed in their study of *homo economicus* theories.

Narratives or discourses are increasingly recognized as structures for reinforcing conceptions, often unconsciously. On a routine basis, we perceive the world through *frames*, which Rein and Schön define as:

“*… a way of selecting, organizing, interpreting, and making sense of a complex reality to provide guideposts for knowing, analyzing, persuading and acting*” (Rein and Schön, 1993).

#### 3.1.4 Openness to prosocial individual behavior

The fourth aspect of recognizing prosociality is to be sufficiently open-minded to notice instances of prosocial individual human behavior. Examples of collaboration and care are not uncommon, and most of us can identify family members or friends who exhibited love and empathy, or someone who exhibited self-sacrificing heroism in life-threatening situations. Everyone’s experience differs, and some may argue that it is more common that people prioritize their own interests rather than social interests, but this paper argues that it is possible to strengthen the capacity for prosociality.

So why is our perception of self-interest so pronounced that some consider it to be predominant in human behavior? One factor may be religious beliefs about original sin, which may be used to justify the status quo and relieve us of the need to change ourselves (Slater, 2006). Another factor could be the perception and influence of leaders. Along these lines, Bregman (2019) concludes that authoritarian leaders assume that citizens will commit crimes, as they themselves would do, particularly in situations of lawlessness during emergencies. Yet, as Bregman points out, evidence from 700 disasters affirms that most behavior in these settings is altruistic, and that crime actually drops.

The evidence for prosociality is further reinforced by findings in biology. For example, Lakoff (2008) explains that brain science research on mirror neuron circuits and associated pathways demonstrate that empathy and cooperation are natural and hard-wired into our biology. We feel what others feel, which can explain why we trust and do things for others. Moreover, in their book, *Prosocial: Using Evolutionary Science to Build Productive, Equitable and Collaborative Groups*, Atkins et al. (2019) demonstrate that a group’s cooperation and morality helps them to be successful in the process of evolution through a multi-level selection.

There are many examples of prosocial coordination among protagonists, and as Hopkins stated: “If we wait for governments, it will be too late. If we act as individuals, it will be too little. But if we act as communities, it might just be enough, and it might just be in time” (Hopkins, 2019, p. 6). In an example from a marginalized Brazilian favela, residents became “agents of their own transformation” through their own “protagonism” and community dialogue rewriting the traditional bureaucratic calculus which waits in vain for government funding or private investment (Boullosa and Peres, 2022). In another example, a woman from Burkina Faso combined indigenous knowledge with science to work with her community to map natural resources and then partner with government in local climate action (Hawken, 2021). The UN *2022 State of the World’s Volunteerism Report* provides many examples of the actions of such protagonists (UN Volunteers, 2022), and the *Water Integrity Global Outlook 2021* report cited the city of La Paz in its anti-corruption campaign which strengthened participation and transparency with citizens (Water Integrity Network, 2021). Examples of collaboration in good governance are captured globally through the Open Government Partnership, such as the accountable procurement procedures and citizen monitoring in Moldova (Open Government Partnership, 2022).

Prosocial behavior can be promoted, as shown in a systematic, multi-year and multi-country experience initiated by Nur University in Bolivia. This program focused on strengthening capabilities such as learning from reflection on action, systematic thinking, creative initiative, love, encouragement, justice, and transformation of patterns of domination. They drew on the work of Mezirow (2000) in a change process which included challenging mental models, transforming understanding through critical analysis, and adopting a new conceptual framework. Their program was implemented with thousands of educators and community workers in South America, and subsequently adapted to health ministry officials within the WHO program Good Governance for Medicines (Anello et al., 2020).

#### 3.1.5 Openness to prosocial collective behavior

The fifth aspect of the faculty for recognizing prosociality is to be open to a more *positive reading of collective human progress*. It is hypothesized that if one recognizes prosocial behavior, then one is more likely to see progress in history, and a negative view of progress is likely to go hand-in-hand with an emphasis on the negative side of human behavior. Rochester (2018) sees humanity as a glass that is both half empty and half full; on the empty side we see the rise of terrorism, clash of civilizations, genocide, and climate change, and on the full side we see unprecedented international cooperation, the absence of major global wars, rising income, reduced infant mortality and illiteracy, democratization, and acceptance of women as full citizens. Rosling (2018) has pointed out the 50% reduction in extreme poverty and 80% of children being vaccinated, facts which most people around the world are unaware of due to the media or their own attention filters. Pinker (2011) cites a significant reduction of child abuse, homicide, and warfare, which has been achieved through the rise of organized systems of government and criminal justice, a human and animal rights revolution, and the presence of educated citizens willing to take government to account. He concludes that while human nature does include violent inclinations, it also has peaceful and cooperative tendencies, the “better angels of our nature.”

### 3.2 Embracing the complexity of human nature

Having established the possibility of prosocial behavior, it is important to accept and embrace the complexity of human nature, as a basis for strengthening people’s proclivity toward collective well-being. This ability can be strengthened by acknowledging:

- that people are motivated by a mixture of motives,
- the importance of social influences,
- the influence of leaders, through the systems they create and their expectations of others, and
- that people’s capacities should be expected to be diverse, and to evolve.

#### 3.2.1 Mixed motives

Understanding that people may have a combination of motives can encourage acceptance and patience with others, and flexibility in adapting to different contexts and evolving development approaches. In this regard, Chin and Benne (1985) draw on behavioral science to write about three views of human nature, with corresponding approaches to influencing behavior. Rational/empirical views assume that people naturally seek to maximize their self-interest and rationally calculate optimal outcomes, rather than being swayed by emotional factors. Power/coercive views assume that people avoid change unless motivated by the threat of penalties or punishments. Normative/re-educative strategies are based on a hypothesis that humans are motivated by personal meaning, habits and values, and their relationship with their environment. All views may have a portion of the truth, considering the diversity among people and across time and different situations. For example, a person may be self-sacrificing within their family or faith community, yet highly competitive in their business setting, and advocating for policies that put forward their own national self-interest irrespective of the cost to humanity. Development programs should therefore be prepared to adopt strategies that combine incentives, penalties, and efforts to strengthen prosociality, while carefully adapted to the needs of different situations. This suggests the need for further research about human nature and its expression in different contexts.

#### 3.2.2 Social influences

The influence of those around us is crucial in conditioning our perceptions and behavior, and the relative importance of agency vs. structure is discussed below. People are influenced by the behavior they *observe* in others, but also by their perception or belief about the behavior, motives, and thoughts of others. Gonzalez de Asis et al. (2009) state that most people are “honest but sinful” in that they want to be honest but sometimes act corruptly if they see that others are doing so. Usually there are a few people who are consistently “devils” or “angels,” but the influence of the former can often tip the balance toward systemic corruption. Spector (2022) also highlights the need to address drivers of behavior at both the group and individual level through a social psychology focus which captures people’s strategies around power relationships, self-control, social norms, and emotions.

Our perceptions about others’ values can shape our vision for our shared society. In an American context, Rose cites a study of 5,200 respondents which asked respondents whether they would define success as: (A) having followed their interests and talents to become the best they could be at what they care about the most, or (B) being rich, having a high-profile career, or being well-known. When asked about their own view of success, 97% of respondents picked answer A, whereas 92% thought that most others would choose B. Further, respondents revealed that the most important attributes for success in their own lives were character, good relationships, and education, even though they believed others prioritized wealth and power. Thus, we misread the values and motives of others, and this can cause us to conform to a “collective illusion” of what we believe others expect of us and this colors our views on key issues such as war, climate change, gender bias, and ethical behavior (Rose, 2022).

Given these examples of social influences, social change interventions should aim to work with all protagonists simultaneously because isolated efforts may have limited effects. Hence, Gonzalez de Asis et al. recommend multi-stakeholder and multi-sector integrity strengthening initiatives. This highlights a key point, that social ideals can either be strengthened or depleted depending on the actions of others, as well as by policies.

Research by economists Ashraf and Bandiera (2017) recognizes prosocial behavior as a form of asset, and they propose formalizing it as altruistic capital. This capital can be accumulated or depleted in the individual through their own altruistic acts, and organizational policies can encourage altruistic behavior or conversely they can deplete altruistic capital if employees feel disconnected from the meaning of their work.

#### 3.2.3 Importance of prosocial leadership

Given the multidirectionality of influences over human behavior, the role of leadership in promoting prosocial values is essential. Transformational leaders inspire followers to transcend their own self-interest for the good of the organization and society, in contrast with transactional leaders who guide and motivate their followers in the direction of established goals (French and Bell, 1995). Atkins et al. (2019) provide a comprehensive program for building collaborative groups, which emphasizes shared identity and purpose, equitable distribution of contributions and benefits, fair and inclusive decision-making, monitoring agreed behaviors, graduated responses to helpful and unhelpful behavior, fast and fair conflict resolution, authority to self-govern, and collaborative relations with other groups.

Leadership can be exercised by the way we think of others, as well. The phenomenon of *self-fulfilling prophecies*—often referred to as the Pygmalion effect—demonstrates how our positive perceptions of others can affect their behavior. Rosenthal and Jacobson released a study in 1968, which demonstrated that teachers’ positive expectations of certain students translated into improved academic outcomes and IQ, research findings which have been replicated over the years in varying national contexts (Szumski and Karwowski, 2019). In workplaces, Veestraeten et al. (2021) cite global Gallup studies that have shown that when employees are more engaged, they have 21% improvements in productivity and 22% improved profit. They also find that optimal engagement is dependent on beliefs by both employees and employers about whether employees are hardworking, productive, and willing to “go above and beyond.” Spanish researchers (Cobos-Sanchiz et al., 2022) found that by positively reinforcing the factors of emotional intelligence (including empathy), education programs and positive community influences can reinforce students’ intrinsic motivation.

#### 3.2.4 Diversity and evolution of prosocial capacity

As discussed above, it would be a mistake to overgeneralize about people’s prosociality, and what is needed is to recognize that each person displays a different mixture of motives, interests, and capacities, and this is often partly shaped by each set of circumstances. Likewise, while we must take the current capacities and practices of these protagonists as a starting point, this should not limit our imagination for future planning, as protagonists’ capacities should ideally continue to grow. The protagonist framework outlined below suggests a simple process to assess each situation and set of stakeholders, as a basis for planning development interventions.

Thus, this paper suggests that while it is essential to recognize the prosociality in human nature, it is likewise important to acknowledge that people are driven by mixed motives, and that various social influences and expectations can shape individual behavior. This provides a basis for taking a balanced approach to engaging people to contribute to collective development efforts.

## 4 Protagonists of development

The preceding sections on participation, capacity strengthening, and prosociality lead to the question of how to think about strengthening these capabilities among all the protagonists of development. Prosocial behavior is partly individual, but it is also manifested by communities and institutions, within webs of relationships, systems, and structures This paper suggests an exploratory analytical framework to facilitate research and action about and by these protagonists, through an analysis of their antecedent knowledge, values, and culture; sense of agency and resources; stance; roles and functions; relationships; and learning practices and approaches. These could be considered as essential characteristics that need to be included in efforts to strengthen capabilities toward more prosocial and effective development. This section concludes with a presentation of this protagonist’s framework, but first it provides a review of other stakeholder frameworks, and then discusses key concepts necessary to introduce the framework.

### 4.1 Protagonists as a framework of stakeholders

One of the challenges of reforms of development practices is that we often lose sight of the complexity of actors and relationships, and either tend to make sweeping platitudes about the participation of stakeholders, or allow our thinking to be constrained by pre-existing conceptual frameworks. This section gives an illustration of several informal framings that have emerged to conceptualize the spaces or levels of stakeholder participation.

The UNDP model of capacity strengthening (UNDP, 2009) analyzes individuals, organizations, and enabling environments, and while this is a positive step forward, these describe different types of phenomena. Capacity strengthening of stakeholders (individuals, organizations) is easy to conceptualize, but we cannot think similarly of the capacity strengthening of spaces (enabling environments). This adds complexity to taking the next step toward analyzing characteristics of stakeholders, so as to move toward theory-building around how they interrelate and can potentially collaborate more effectively.

**Figure 1:**
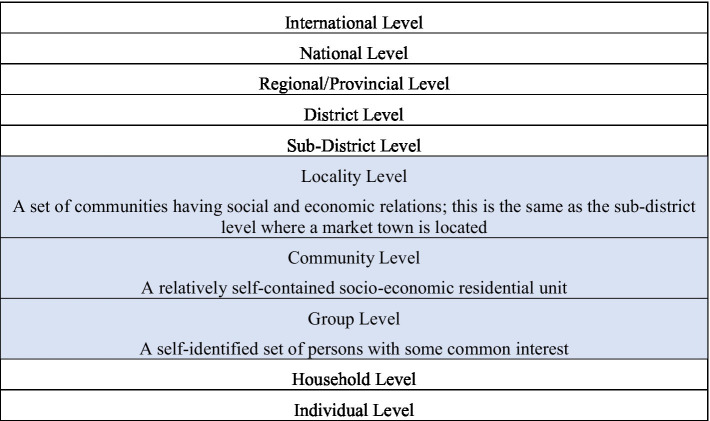
Levels of stakeholders (adapted from Uphoff, 1992).

Representing stakeholders in geographical levels or scales is a common metaphorical approach. Uphoff (1992) provides one such breakdown (Figure 1). One possible issue with these hierarchical types of models is that they may unconsciously imply hierarchies of importance. Another concern is that at times they may prematurely congeal thinking of capacity strengthening and functioning into categories based on geographic variables, while overlooking other variables. For example, one could instead group organizations into formal vs. informal organizations, or government vs. business vs. non-profit organizations. It is suggested that many of these geographic models emphasize the status quo of governance, while de-emphasizing relations and social structures that cross geographic lines.^3^

The policy network approach is another way of thinking about stakeholders, which highlights the way that a variety of state and non-state actors contribute to formulating policy through their membership in policy discourses, the resources they bring to bear, and the way they are integrated through interactions and shared values (Rhodes and Marsh, 1992). Many have pointed out that the existence of a network does not provide an explanation of the interaction of these stakeholders nor explain how policies change, and this has led to various evolutions in this approach (Rhodes, 2017), such as integrating networks with concepts of interests, ideas, and institutions (Shearer et al., 2016). Systems theory can provide another perspective on the interaction of human systems with ecosystems, breaking systems down into elements, interrelations between them, and the function or purpose of each element (Meadows, 2008).

All of these models partly inform the present protagonist framework, which posits some key characteristics of each protagonist which can shape the analyses of their capacity strengthening and participation. Such an analysis would be valuable in programs promulgated by development agencies to increase stakeholder participation in support of achieving the SDGs.^3^ A protagonist analysis process could be as follows: (1) identify the relevant protagonists; (2) understand their characteristics (see below); (3) assess and help strengthen their capacities; and (4) support and monitor their participation. While the use of this approach by existing development agencies may be one clear application, it should be noted that any protagonist may equally follow such a process to view themselves within a network and determine how to work with other stakeholders.

Visualizing stakeholders as protagonists is a practical framework which is informed by critical political economy analyses of inequities, feminist views of entrenched patriarchy, and a reading of racial and nationalistic conflicts. By referring to a set of social categories such as individuals and institutions, the intention is not to affirm a structural-functional view of society with stable relationships or an implied bias toward the status quo. On the contrary, the analysis which a social-conflict approach (Macionis and Gerber, 2011) provides can help empower the marginalized and transform development efforts, and this is what the framework of protagonists intends. The framework also seeks to bring the local and global together in a learning-oriented and capacity strengthening approach which can balance agency and structure. It builds on the work of Karlberg and Smith (2021) who discuss the importance of individuals, communities, and institutions as the three sets of protagonists of community-building and social change in their discussion of the experiences of the Bahá’í international community.

### 4.2 Concepts inherent in the protagonist framework

Having introduced protagonists as a way to think about stakeholder engagement, it is necessary to provide some working definitions for the concepts of protagonist, individual, community, and institution. The ideas of structure and agency, stance, and learning are also of central importance in contributing toward a gradually improving conceptual framework for prosocial protagonists.

*Protagonist* is the word proposed here, which is closely related to the terms stakeholders or actors, but it implies a sense of active ownership and engagement. The most common meaning of protagonist in English is the leading character in a story, and this meaning applies if we think about writing narratives of social change. It also implies someone who is actively promoting something, rather than opposing something, as in *antagonist.* In Spanish, the corresponding term *protagonista* is commonly used, and one example of this usage is a paper that describes how civil society organizations can be *protagonistas* of governance and local development in Mexico (Ramirez Casillas, 2008). A program in Argentina helps youth *protagonistas* to reconnect with their personal power and purpose and transform into the best version of themselves, working in four domains of language, bodies, emotions, and the spirit (Protagonista de Cambio, 2022).

*Individuals* can be taken as a starting point of discussions of protagonists because it is as individuals that we think and act, and it is relatively well-understood that individuals can become empowered and take initiatives. Individuals compose communities and institutions, but the converse is not true: institutions and communities do not *physically* become part of an individual. Having said that, communities and institutions do exert influence through culture and structures, and they condition the thinking and behavior of individuals to such an extent that they essentially become integrated into the consciousness of the individual. According to the Frankfurt School of critical social theory, for example, people are essentially social and constituted by intersubjective relations, rather than being defined by a narrow self-identity (Calhoun, 1996). Hence, development programs often seek change within individuals or institutions, but quite commonly the final impact is expected to be measured in the lives and perceptions of individuals. A focus on individuals also leads to disaggregating and being more attentive to the inclusion of various marginalized groups. Individual inclusion is partly determined by power relations among group members, but initiative is also needed on the part of the individual participants, which raises the issue of structure and agency that is discussed below.

*Institutions* could be taken as all types of formal organizations including public, private, non-governmental or non-profit organizations (Parsons, 2005). The line between community and institution here should be taken as a fuzzy boundary, subject to contextualization and modification over time. Institutions are understood here in the *organizational* sense of the word, in keeping with common development discourse of institutional strengthening and local and global institutions. Committees at the community level which are formalized, such as an elected development committee, can be considered as institutions in this framework. Institutions can be protagonists of development, and they can either operate in a prosocial manner or in a way to protect their own bureaucratic interests or maximize the interests of their constituency as opposed to promoting the wellbeing of humankind. It is probably accurate to say that there is generally a mixture of motives and objectives in how institutions operate.

*Communities* also have a role in shaping people’s thinking and action and influencing institutions, and they are entities which are distinct from any one individual member or associated institutions. Communities are informal groupings of people with strong social ties, shared interests, and history, often geographically proximate but increasingly formed through shared activities in physical or virtual spaces. Communities can be protagonists in the sense that most development initiatives work with groups of people, and empowerment and a sense of ownership tend to occur collectively in truly participatory processes. Many types of development would not take place if not for community efforts, for example: group savings and lending, groups of mothers promoting health, men’s groups in support of gender equity, farmer groups that share knowledge on sustainable agriculture measures, and communities that resolve to eliminate “open defecation” and establish village-wide hygienic conditions. Rose (2022) asserts that humans are profoundly social, and our desire to align with others can be considered a “conformity bias” which is hard-wired into our biology. A key feature of communities is the way they influence (and are influenced by) the thinking of various individuals, and share a similar mutual influence with institutions. Community groups also may take development initiatives and evolve into institutions.

*Agency* is a crucial concept in setting out a framework of participants in governance, which Kabeer defines as: “the ability to define one’s goals and act upon them … the meaning, motivation and purpose which individuals bring to their activity, ‘the power within’” (Kabeer, 1999). Power and resources of various kinds (financial and human resources, expertise, and contacts) could arguably be included as conferring agency. Knowledge can also confer agency, e.g., an individual’s knowledge of their human right to participate, and the knowledge by other stakeholders of this human right. The intention of this framework is to focus on the actors that bring that sense of agency to initiate and take ownership of the development process while acknowledging that these actors are simultaneously enmeshed in social structures and relations.

**Figure 2:**
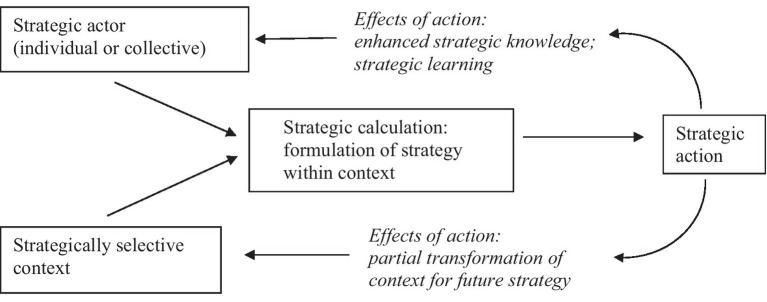
Interplay of agents and structure. © (Hay, 2002), Political Analysis: A Critical Introduction, Red Globe Press, used by permission of Bloomsbury Publishing Plc. All rights reserved.

*Structures*, or “those overarching systems of social relationships that stand as seemingly external forces determining the lives of individuals” (Holton, 1996), are clearly important as a counterpoint to agency. Arguably, structures and human agency jointly set the parameters for social change even while they modify each other through action. Thus, the supposedly objective roles, responsibilities, and relationships among these protagonists need to be freshly considered in the light of their strong interrelatedness and evolution through ongoing actions. Drawing on post-empirical social science helps to avoid an objectivist assumption that objects (e.g., society, structures) predominate over the subject (e.g., knowledgeable human agent). Giddens’ structuration theory aims to reconceptualize this as a “duality” of interdependent components, where neither agent nor structure can exist without the other, while also seeking to avoid an exaggerated emphasis on subjectivism (Giddens, 1984). Hay and Jessop assert that structure and agent are not just like flip sides of the same coin, as others have argued, but more like the alloy metals which appear completely fused in the coin. Figure 2 illustrates the interplay of agents (individual or collective actors) and structure (contexts) as they formulate and carry out strategic actions. As a result of their action, the agents learn, and the structure is partly changed (Hay, 2002). Again, the term “context” as used by Hay and Jessop could be considered as the structure, or more specifically, a geographical and ideational space which included communities and institutions.

In a more accessible sense, individuals, communities, and institutions influence each other through such means as community peer pressure on individuals, government incentives on individual behavior, or community mobilizations against government policy. Another caution is needed about objectivity, because the “knowledge” of both observers and participants is at least partially socially constructed. Hence, as previously discussed, it is important to surface the frames that practitioners and researchers hold about the nature of these protagonists. Part of empowering protagonists would surely be to use participatory research approaches, possibly drawing on phenomenological methods to prioritize participants’ lived experiences in interpreting meaning (Creswell, 1998).

On this point, *stance* is a helpful concept, and for purposes of this paper it can be defined as a general orientation toward others, particularly our assumptions about the characteristics of other people including their knowledge and motives, and the way we interact, take joint action, and learn with others. The idea of stance has been used in the context of experiential learning (Salmon et al., 1990; Hutton, 1990) to imply a set of attitudes, values, beliefs, and expectations, as well as the position from which one views an action. This paper suggests that prosociality could be seen as a stance, as it is a generalized orientation that is based on beliefs about human nature, and it requires an ability to recognize prosocial behavior while working effectively with mixed motives. The way we see our own prosociality generally mirrors to some extent the way we regard others and make assumptions about their motives, which is important to facilitate a more prosocial emphasis in action and also in research, as discussed above.

For the analytical purposes of this paper, values and culture are categorized separately, while stance is taken as including only those specific attitudes, values, and beliefs that determine how the actor orients themselves toward other actors. Feminist standpoint theories provide another complementary way to think about stance in recognizing that one’s social and historical place in the world determines the way that one sees the world, and that therefore women or other marginalized individuals are more able to question the status quo and see the true nature of the world and its relations (Bowell, 2022).

Stance is a characteristic of how an actor generally approaches situations, and a key issue is whether one takes a stance of discovering the knowledge and stance of others, or mainly a stance of wanting to confirm one’s own knowledge and self-image (Vuille et al., 2013). A stance of knowing does not provide any opening for new information to come in and reach our brain, and as a consequence does not allow new learning (Theiopoulou, 2024). Stance may be helpful in fleshing out the way that protagonists see their positionality among networks of stakeholders, how they regard others and interpret their motives, as well as their willingness to learn and collaborate. *Learning* is a subject that has been relatively well analyzed in the context of development monitoring, evaluation, and learning approaches, with multiple resources of this type curated by the Food Security and Nutrition network of US NGOs and shared at their website.^4^ An effective learning approach is needed not only to guide the strategic actions undertaken by the protagonists but also to reflect on and modify the roles and relationships among them.

### 4.3 Elements of a prosocial protagonist conceptual framework

With the benefit of these working definitions and preparatory ideas about stakeholder frameworks, this section provisionally outlines elements of a conceptual framework for building the capacities and engaging the participation of protagonists in support of global development.

**Figure 3:**
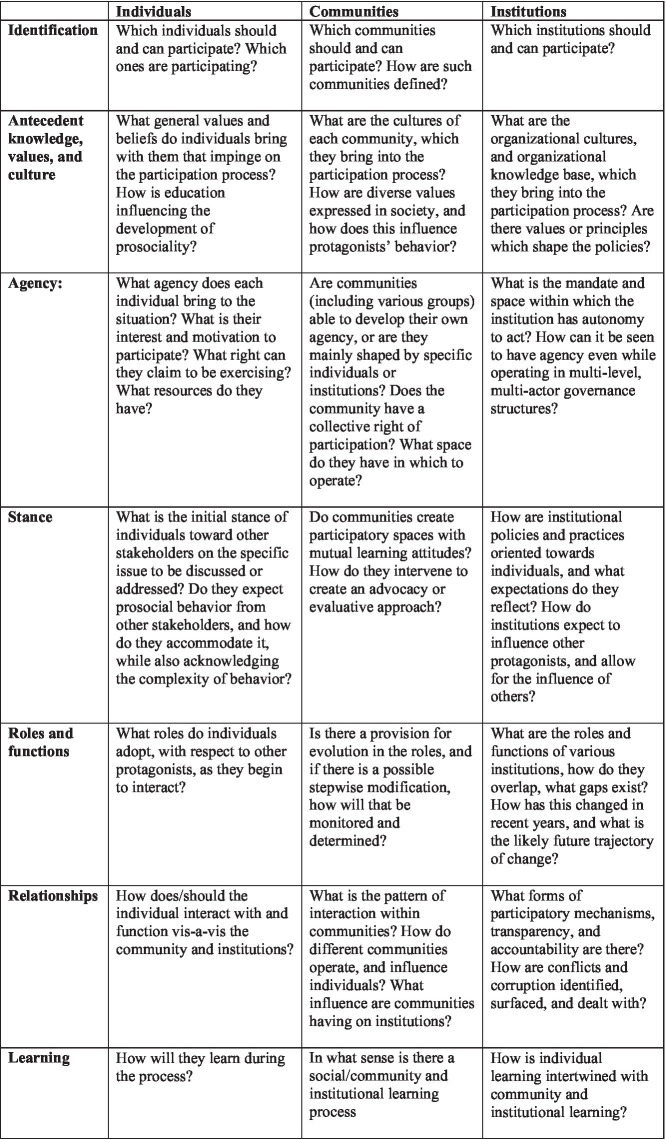
Prosocial protagonist framework matrix.

The framework is in the form of a matrix which poses a series of questions to help to analyze the main characteristics of each protagonist in a given development context. These questions were created simply from a reflection on the interaction of actors in development, their strengths and weaknesses, and on the potential for prosocial collaboration (Figure 3). This framework is exploratory in nature, and undoubtedly others will modify and elaborate it, ideally in the light of more evidence and insights from research and practice.

## 5 Discussion

All of this leads to a framework of prosocial protagonists… an idea whose time has come. This unique historical juncture of crises and heightened capacities and opportunities should be seen as presenting a “new world,” requiring a “new enlightenment,” and a “new social contract for a new era.” The multiplying crises facing humanity, such as climate change, pandemics, and war, demand a transformational response. Participation of the protagonists of development is an essential goal of the international community, and much more value needs to be derived from this, by strengthening their capacities to help achieve development goals, which are embodied in the SDGs. This paper provides elements of a working conceptual framework of three categories of *protagonists* in global development: individuals, communities, and institutions. There is an inescapable integration and mutual influence between these protagonists, and the nature of these influences depends on many factors, which this framework can help to elucidate.

It needs to be stressed here that humanity needs to be proactive rather than reactive, willing to engage in participatory reflection on our basic objectives and approaches rather than just modifying actions to meet the same objectives. Those from wealthy nations have a significant role today in supporting the autonomous capacities of protagonists in the Global South, taking responsibility for their inordinate impact on the environment, and creating space for economic systems to evolve toward greater sustainability and equity. And yet the protagonist framework—and the focus of this paper—requires focusing more on those in the Global South in order to stimulate improved approaches to international development, being open to different types of participation emerging over the coming years. It is important to strengthen capacities alongside this, while avoiding making presuppositions or limiting their roles according to current governance arrangements and apparent capacities. The idea of prosocial protagonists calls for change agents that willingly and synergistically collaborate with a range of stakeholders to contribute to the common interests of humanity—in this instance, to the SDGs. The international community needs to recognize and support more of the examples of prosocial behavior of protagonists, which will be essential to the long-term interests of humanity. At the same time, it is important to be realistic and patient with the persistence of short-term and self-interested attitudes and practices. Severe injustice and corruption are still commonplace, and both incentive-based and punitive policies are needed, supported by active engagement of protagonists in insisting on transparency and helping hold others accountable. The prosocial potential exists, even if at times this light becomes dimmed, and it is important to demonstrate trust and have an expectation of prosocial behavior, while allowing protagonists the space and freedom to develop their capacities.

If one accepts that prosocial motives and behavior are inherent in the human condition, there seems to be no defensible option not to fully support efforts to educate, encourage, and promote it. The only logical reason that powerful actors would not choose to do so is if they preferred to maintain a myth that humans are incorrigibly selfish, in order to justify their own advantaged position within the status quo. Education and promotion of prosocial behavior in schools, workplaces, institutions, and communities are crucial, with the help of moral, ethical, spiritual, and cultural teachings and any other means that have evidence of results. This should also include moves toward coordination on policies around issues of encouraging and supporting volunteers, and promoting systemic integrity to combat corruption.

In summary, the development of a prosocial protagonist framework, elements of which are included here, holds the promise of:

- Engaging a more prosocial participation of all stakeholders, while recognizing the complexity of human nature and taking account of the need to work with the current prevalence of self-interested behavior.
- Coordinating approaches to strengthening capacities of the various stakeholders that can play roles in facilitate broader participation.
- Giving greater attention to the grassroots level by highlighting the agency and participation of individuals, communities, and local institutions.
- Analyzing and working with relatively powerful political and economic institutions to help them learn and be held accountable to more effectively contribute to social goals.
- Providing a conceptual basis to talk about changing dynamics and capacities among protagonists, such as the evolution in the types of communities that are significant in people’s lives, or the growing capacity of institutions at the very local level.

Some readers may find the approach proposed here to be idealistic, and alternative proposals are welcome. But the question remains: is it not naïve to think that by continuing to work in the same way, we will somehow achieve the SDGs within the next 6 years, much less cope with other unexpected eventualities or crises that will arise?

## Data Availability

The original contributions presented in the study are included in the article/supplementary material, further inquiries can be directed to the corresponding author.

## Footnotes

^1^This paper was written in response to a call for papers to apply social theory to the task of strengthening capacities toward achieving the Sustainable Development Goals. While there will be different opinions about how effective or inclusive the SDGs are, they can still be regarded as the central representation of humanity’s development aspirations.

^3^It should be acknowledged that this is not the only context for development, but such efforts and the SDGs are the reference framework for this paper.

^4^ https://fsnnetwork.org
